# Capture Hi-C reveals novel candidate genes and complex long-range interactions with related autoimmune risk loci

**DOI:** 10.1038/ncomms10069

**Published:** 2015-11-30

**Authors:** Paul Martin, Amanda McGovern, Gisela Orozco, Kate Duffus, Annie Yarwood, Stefan Schoenfelder, Nicholas J. Cooper, Anne Barton, Chris Wallace, Peter Fraser, Jane Worthington, Steve Eyre

**Affiliations:** 1 Arthritis Research UK Centre for Genetics and Genomics, Centre for Musculoskeletal Research, Institute of Inflammation and Repair, Faculty of Medical and Human Sciences, Manchester Academic Health Science Centre, The University of Manchester, Stopford Building, Oxford Road, Manchester M13 9PT, UK; 2 Nuclear Dynamics Programme, The Babraham Institute, Cambridge CB22 3AT, UK; 3 JDRF/Wellcome Trust Diabetes and Inflammation Laboratory, Department of Medical Genetics, NIHR Cambridge Biomedical Research Centre, Cambridge Institute for Medical Research, University of Cambridge, Wellcome Trust/MRC Building, Cambridge Biomedical Campus, Cambridge CB2 0XY, UK; 4 NIHR Manchester Musculoskeletal Biomedical Research Unit, Central Manchester Foundation Trust, Manchester Academic Health Science Centre, Oxford Road, Manchester M13 9WL, UK; 5 MRC Biostatistics Unit, Cambridge Institute of Public Health, Forvie Site, Robinson Way, Cambridge Biomedical Campus, Cambridge CB2 0SR, UK

## Abstract

Genome-wide association studies have been tremendously successful in identifying genetic variants associated with complex diseases. The majority of association signals are intergenic and evidence is accumulating that a high proportion of signals lie in enhancer regions. We use Capture Hi-C to investigate, for the first time, the interactions between associated variants for four autoimmune diseases and their functional targets in B- and T-cell lines. Here we report numerous looping interactions and provide evidence that only a minority of interactions are common to both B- and T-cell lines, suggesting interactions may be highly cell-type specific; some disease-associated SNPs do not interact with the nearest gene but with more compelling candidate genes (for example, *FOXO1*, *AZI2*) often situated several megabases away; and finally, regions associated with different autoimmune diseases interact with each other and the same promoter suggesting common autoimmune gene targets (for example, *PTPRC*, *DEXI* and *ZFP36L1)*.

## Abstract (web summary)

There is evidence that a proportion of the polymorphisms identified by genome-wide association studies lie in enchancer regions. Here the authors use Capture Hi-C to investigate the interaction with targets in autoimmune disease, showing interactions can be long range and cell-type specific.

## Graphical abstract

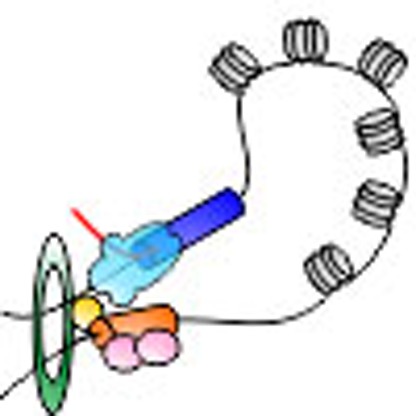

The idenfication of the precise gene targets of variants associated with complex traits detected through genome-wide association studies (GWAS) has proved challenging^1^ but is essential if the full potential of genetic studies is to be realised. Accumulating evidence suggests the majority of these variants lie outside traditional protein-coding genes and are enriched in enhancer regions, which are both cell-type and stimulus specific^2^,^3^,^4^. The task now is to identify which genes are implicated and understand which cell types are involved, to ascertain the biological pathways that are perturbed in individuals who are genetically susceptible to disease. It is well-established that enhancers regulate gene transcription by physical interactions^5^. These can operate over large genetic distances, so the tradition of annotating GWAS hits with the closest, or most biologically plausible gene candidate, may prove misleading and result in expensive, time consuming efforts to define the function of non-causal genes.

The utility of chromosome conformation capture technology (Capture Hi-C) to detect the patterns of interactions between chromosomal regions has been demonstrated^6^,^7^,^8^,^9^. Here, for the first time, we used this approach to characterize the interactions of confirmed susceptibility loci for four autoimmune diseases: rheumatoid arthritis (RA), type 1 diabetes (T1D), psoriatic arthritis (PsA) and juvenile idiopathic arthritis (JIA) with the aim of linking disease-associated SNPs with disease-causing genes. Uniquely, we have tested the interactions in two complementary experiments: first, Region Capture targets regions associated with disease^10^,^11^,^12^,^13^,^14^; second, Promoter Capture provides independent validation through capturing all known promoters within 500 kb of lead disease-associated single nucleotide polymorphisms (SNPs). Our study expands on recent applications of the Capture Hi-C method firstly, by increasing the depth of sequencing and therefore the resolution, (average 10,000 interactions per restriction fragment), second, by comprehensively targeting the full known genetic component of four related autoimmune diseases and finally by performing complimentary experiments, such that we target the disease-associated regions and, in separate experiments, all gene promoters within 500 kb, so providing direct, independent, reciprocal validation for each interaction. All experiments were performed in human B (GM12878) and T (Jurkat) cell lines, selected because they are most relevant to these diseases^3^. Hi-C libraries were generated for both cell lines^15^, then hybridized to custom biotinylated RNA baits and sequenced on an Illumina HiSeq 2500. We tested for significant interactions using a negative binomial distribution as described previously^6^, performing all experiments in duplicate.

Our findings provide compelling evidence that disease-associated SNPs, currently nominally assigned to the closest plausible gene candidate, may well-regulate genes some distance away. We also show that in a subset of risk loci, SNPs associated to different autoimmune diseases physically interact with and may well-regulate the same genes but with differing enhancer mechanisms. A number of the interactions also show evidence of cell-type specificity, occurring in either the B- or T-cell lines only.

## Results

### Summary of identified interactions

**Figure 1:**
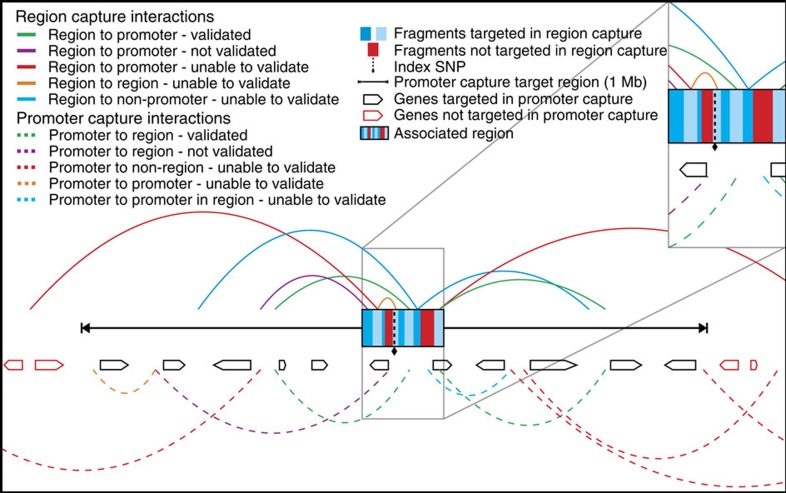
A schematic of a hypothetical associated region including possible chromatin interactions. Chromatin interactions are shown by arcs, those above the promoter capture target region are observed in the ‘Region Capture' experiment; those below are observed in the ‘Promoter Capture' experiment. All potential chromatin interactions are shown and are coloured by their potential to appear and be validated in both capture experiments. Those in green are observed in both the ‘Region Capture' and the ‘Promoter Capture' and comprise the ‘confirmed' interaction set. Interactions shown in purple are only present in one capture experiment and were therefore not validated. Other interactions (red, orange and blue) would only be observed in either the ‘Region Capture' or ‘Promoter Capture' and could therefore not be validated as described. The inset shows a magnified view of the associated region (as defined by LD) detailing which restriction fragments were targeted in the ‘Region Capture' and which were excluded as they appeared in the ‘Promoter Capture'.

**Figure 2:**
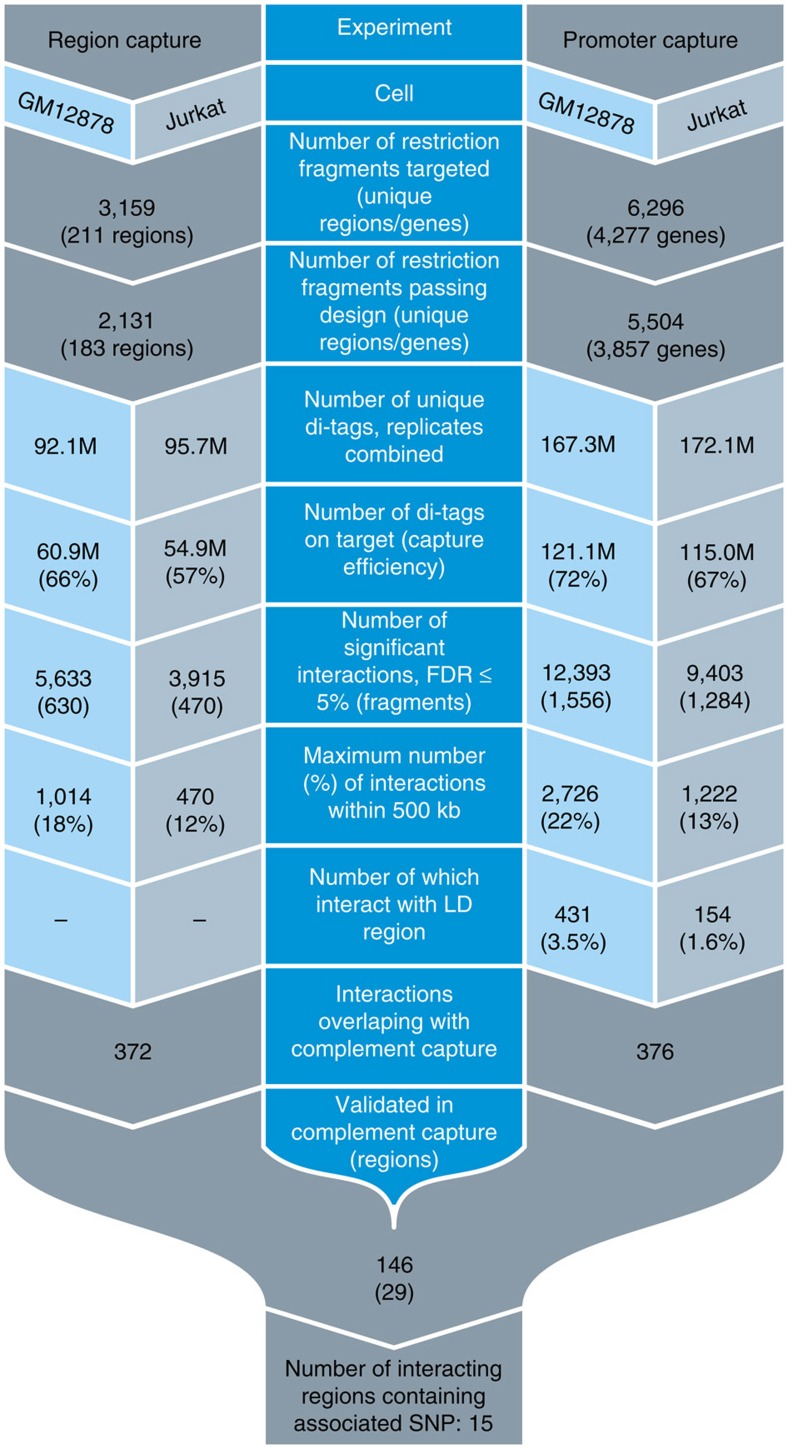
Flowchart summarizing capture Hi-C experiments by cell line. The ‘Region Capture' experiment is shown on the left and the ‘Promoter Capture' experiment on the right. Flowchart sections are coloured by cell type: light blue—GM12878 cells; light grey—Jurkat cells and grey—both cell types. Each section label is shown in dark blue.

Our unique study design determined a complex array of interactions between disease-associated regions and promoters (Fig. 1). After quality control, in the Region Capture experiment, 60.9 million and 54.9 million unique di-tags (comprising one restriction fragment from a capture target region and its ligated interacting partner) were on-target for GM12878 and Jurkat cell lines, respectively (average 21,170 reads per HindIII restriction fragment; 62% capture efficiency). Similarly, in the Promoter Capture experiment, 121.1 million (GM12878) and 115 million (Jurkat) unique di-tags were on-target (average 21,448 reads per HindIII restriction fragment; 70% capture efficiency) (Fig. 2).

**Figure 3:**
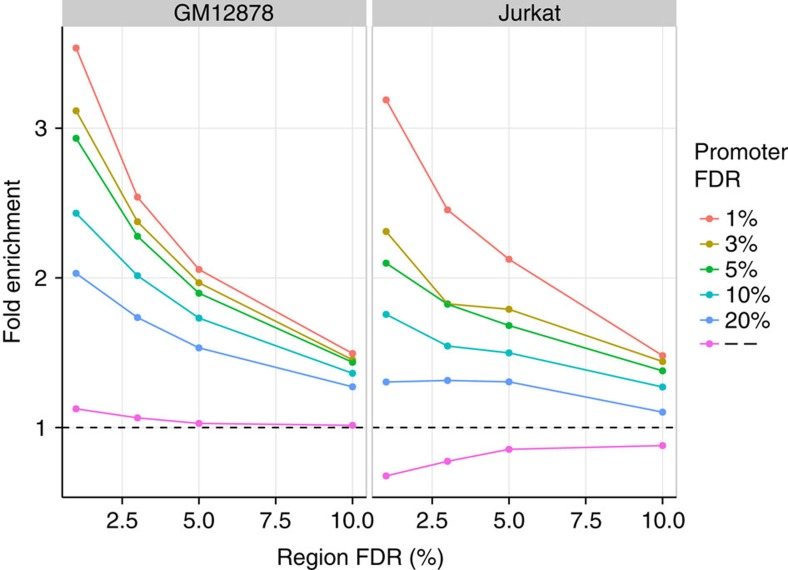
Fold enrichment. Fold enrichment of retained interactions called in the promoter capture experiments with decreasing FDR thresholds, given they had been called in the region capture experiments at the FDR threshold shown. ‘—' shows the enrichment found by focusing only on interactions called in the region capture experiments for which the other end lay in a HindIII restriction fragment targeted in the promoter capture design.

At any given false discovery rate (FDR) threshold, interactions are called with an unknown rate of false negatives. With the assumption that interactions called in both the Region and Promoter Capture experiments are more likely to be true positives compared with those only seen in one experiment, we evaluated several potential FDR thresholds (Fig. 3). We saw a consistent enrichment in interactions called in both experiments at decreasing Promoter Capture experiment FDR thresholds, providing confidence that they represent true interactions. At 5% FDR, we called 8,594 interactions in the Region Capture experiment representing 764 targeted HindIII restriction fragments. Of these interactions 372/8,594 (4.3%) from 116 targeted HindIII restriction fragments demonstrated evidence of interacting with a promoter within 500 kb, and so could be validated by the complementary capture method. Of these, 146/342 interactions were identified in the Promoter Capture experiment (Fig. 2), implicating 29 regions, of which 15 contain disease-associated SNPs (Supplementary Table 1). The majority of significant interactions were cell-type specific, with only 20% found in both cell lines.

We compared our data with publicly available chromatin interaction data in similar cell lines and could detect the well-established interactions with the *cis*-acting regulatory region of the *HBA* locus^16^ (Supplementary Fig. 1a) and interactions in the 5C ENCODE (https://www.encodeproject.org/)^17^ experiments at two regions: *IFNAR1* and *IL5* (Supplementary Fig. 1b,c).

### Interactions with novel candidate genes

**Figure 4:**
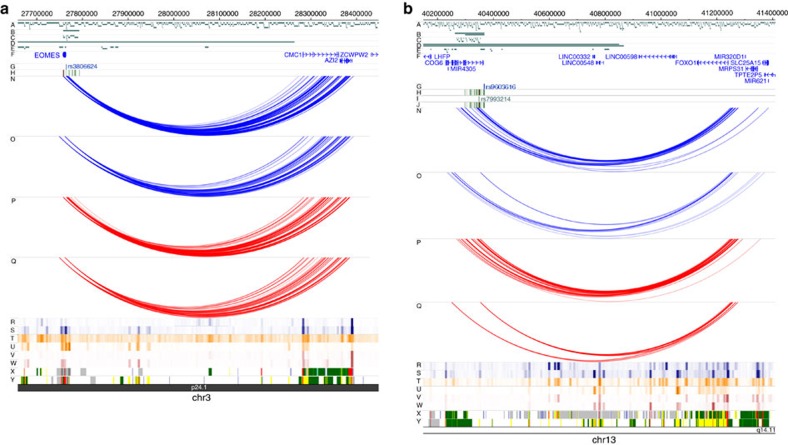
Examples of chromatin interactions implicating novel gene candidates. (**a**) *EOMES* SNPs—both GM12878 and Jurkat cell lines show that SNPs situated proximal to the *EOMES* gene interact with the promoter of *AZI2I*, involved in NFκB activation, situated ∼640 kb away. (**b**) *COG6* SNPs—interactions are shown that link SNPs within the *COG6* to the *FOXO1* promoter, over 1 Mb away, in both cell types. Genomic co-ordinates are shown along the top of each panel and tracks are labelled A–Y (empty tracks removed for clarity): (A) HindIII restriction fragments; (B–E) Regions targeted and restriction fragments included in the region (B,C) and promoter (D,E) capture experiments; (F) RefSeq Genes from the UCSC Genome Browser, downloaded 1 January 2012; (G,I,K) Index SNPs identified for RA (G), JIA (I) and PsA (K). Associations in red were identified in the RA Immunochip study. SNPs in blue were novel associations identified in the RA *trans*-ethnic GWAS meta-analysis, JIA and PsA SNPs were identified in the JIA and PsA Immunochip studies; (H,J,L) Density plots showing 1000 Genomes SNPs in LD (*r*^2^≥0.8) with the index SNPs (green–red) for RA (H), JIA (J) and PsA (L); (M) T1D Credible set SNPs identified in the T1D Immunochip study; (N–Q) Significant Interactions identified in the region and promoter capture experiments in GM12878 (N,O) and Jurkat (P,Q) cells; (R–Y) Data from the WashU Encode track hub showing DNaseI HS sites, H3K4me1 histone marks and H3K27ac histone marks for GM12878 (R,T,V) and CD3 Primary (S,U,W) cells and BROAD ChromHMM states for GM12878 (X) and CD4 Naive Primary cells (Y).

Confirmed interactions provided examples of disease-associated SNPs that do not interact with the nearest gene, but rather with promoters some distance away, implicating entirely different target genes. For example, strong evidence was found to suggest that regions with SNPs associated with RA, situated proximal to the *EOMES* gene, make strong physical contact with the promoter of *AZI2*, a gene involved in NFκB activation, some 640 kb away (Fig. 4a) in both GM12878 and Jurkat cell lines. In addition, variants associated with RA and JIA in the 3′ intronic region of *COG6,* a gene encoding a component of Golgi apparatus, show interactions with the promoter of the *FOXO1* gene, mapping over 1 Mb away, in both cell types (Fig. 4b). Recent findings suggest that the *FOXO1* gene is important in the survival of fibroblast-like synoviocytes (FLS) in RA^18^ and is hypermethylated in RA FLS compared with osteoarthritis FLS^19^, providing strong supporting functional evidence as to gene candidature.

### Common interaction targets mediated by multiple genetic loci

**Figure 5:**
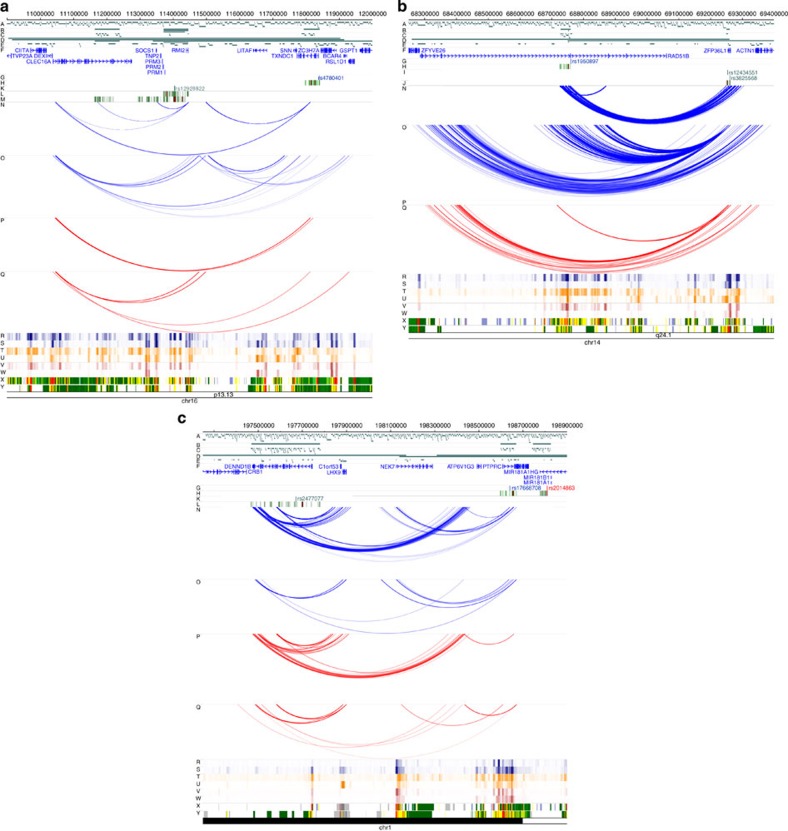
Examples of chromatin interactions linking several disease associations to a common promoter. (**a**) *DEXI*—both GM12878 and Jurkat cell lines show that SNPs associated independently with RA, PsA and T1D interact with the *DEXI* promoter. In addition, evidence suggests that the RA and JIA SNP regions interact in GM12878 cells. (**b**) *RAD51B*—RA associations located within a strong enhancer are shown to interact with the promoter of *ZFP36L1*, a gene involved in B-cell transition, which also contains SNPs associated with JIA. (**c**), *PTPRC*—Variants associated with PsA, within the *DENND1B* are shown to interact with *PTPRC*, a region independently associated with RA. Genomic co-ordinates are shown along the top of each panel and tracks are labelled A—Y as in Fig. 4.

Perhaps the most striking finding comes from genetic regions that harbour susceptibility loci for different autoimmune diseases, where the lead disease-associated SNP for one disease maps some distance from the lead disease-associated SNP for other autoimmune diseases; previously, using the ‘nearest candidate gene' annotation method, different genes would have been assigned to the diseases but our work shows that they may all act on the same gene promoter. We provide three examples below to illustrate the findings. First, the 16p13 region contains SNPs associated with both T1D and multiple sclerosis that locate within intron 19 of the *CLEC16A* gene. A physical interaction between a 20-kb region of *CLEC16A* and the promoter of *DEXI* has previously been reported^20^, although was not detected in the current study. Our data suggest that a separate, independent region, associated with both T1D and JIA, near the *RMI2* gene and 530 kb from the *DEXI* gene, also interacts with the *DEXI* promoter (Fig. 5a). Furthermore, a region proximal to the *ZC3H7A* gene, associated with RA susceptibility, some 1.2 Mb from *DEXI*, interacts with both the T1D/JIA-associated region and the *DEXI* promoter.

The second example is provided by RA-associated variants mapping within a strong enhancer region intronic of *RAD51B*, where a significant interaction is observed with the promoter of the *ZFP36L1* gene. SNPs in the promoter region of *ZFP36L1* are independently associated with JIA but not RA; however, the interaction of the *ZFP36L1* promoter with the RA-associated SNPs suggests that the causal gene in both diseases may be *ZFP36L1* and not *RAD51B*. *ZFP36L1* is a zinc finger transcription factor involved in the transition of B cells to plasma cells and it is noteworthy that the interaction with the RA-associated region was only seen in the B-cell line (Fig. 5b).

Finally we show evidence that SNPs associated with PsA within the *DENND1B* gene make strong contact with a region associated with RA within the *PTPRC* gene, which is responsible for T- and B-cell receptor signalling and maps over 1 Mb away (Fig. 5c).

We, like others^8^,^9^, have demonstrated a complex relationship between promoters and enhancers, where promoters interact with many enhancers and enhancers interact with many promoters, rarely in a one-to-one relationship (Fig. 1 and Supplementary Table 2). Enhancers containing risk variants for autoimmune diseases can, therefore, ‘meet' at the same promoters. This challenges the assumption that disease-associated SNPs have to be in close linkage disequilibrium (LD) to have a disease related effect on the same gene. In addition, these findings may well-suggest an evolutionary phylogeny, where polymorphic variants regulating expression of the same gene result in either different autoimmune diseases or different molecular mechanisms resulting in risk of the same disease.

### Interactions with previously implicated loci

Among the other 141 confirmed interactions, we observed examples of disease-associated SNPs within the 3′ untranslated region, or within introns of a gene, interacting with the promoter of the same gene (*STAT4*, *CDK6,* Supplementary Fig. 2a,b); disease-associated SNPs within lncRNA interacting with the promoter of genes (*RBPJ,* Supplementary Fig. 3) and several examples of restriction fragments, proximal to those containing disease-associated SNPs, interacting with promoters some distance away (*ARID5B*, *IL2RA*, *TLE3,* Supplementary Fig. 4a–c), supporting recent findings that disease-associated SNPs are enriched outside transcription factor-binding sites^3^.

### Long-range interactions

Perhaps unexpectedly, ∼80% of significant interactions occurred at distances exceeding 500 kb (Supplementary Fig. 5) and interacted with ‘non-promoters', reducing the number of interactions available for co-validation in the Promoter and Region Capture experiments (targeted genes in the Promoter Capture not extending that far) and reinforcing the idea that GWAS regions may be involved with complex long-range gene regulation possibly involving multiple enhancer elements. To investigate whether these are likely to be true interactions, we compared results from the largest Hi-C data set on GM12878 cells reported, to date^21^. Of the 4,607 longer distance interactions (>500 kb) we called at FDR <5% in our data, 377 were found at 50 times observed over expected in the independent Hi-C data set (Supplementary Data 1). This provided both strong confirmation of our long-range capture Hi-C results we already co-validated with Promoter and Region Capture (for example, *FOXO1*, *ZFP36L1*, Supplementary Fig. 6) and supports many potentially novel interactions (for example, *MMEL1*, Supplementary Fig. 7), but detailed examination to confirm these long-range interactions is now required.

## Discussion

Our targeted Capture Hi-C analyses have identified, for the first time, many long-range interactions between autoimmune risk loci and their putative target genes. Using this methodology we have intriguing data illustrating that regions associated with more than one disease, often some distance apart, interact with the same gene and that associated regions can ‘skip' genes to interact with more distant novel candidates. Our results provide new insights into complex disease genetics and changes the way we view the causal genes in disease, with obvious implications for pathway analysis and identification of therapeutic targets. Since we uncovered evidence of cell-specific interactions, the current study is likely to be only the beginning of similar explorations. Further work to characterize functionally the observed interactions, including eQTL studies using a range of cell types and stimulatory conditions, are required to determine how disease-associated SNPs influence the risk of disease, with the aim of better understanding disease aetiology.

## Methods

### SNP and region associations

All independent lead disease-associated SNPs for RA were selected from both the fine-mapped Immunochip study^10^ and a *trans*-ethnic GWAS meta-analysis^11^. Lead disease-associated SNPs were also added from the Immunochip fine mapping studies for JIA^13^ and PsA^12^. This resulted in a total of 242 distinct variants associated with one or more of the three diseases after exclusion of *HLA-*associated SNPs. Associated regions were defined by selecting all SNPs in LD with the lead disease-associated SNP (*r*^2^>=0.8; 1000 Genomes phase 1 EUR samples; May 2011). In addition to the SNP associations, credible SNP set regions were defined for both T1D- and RA-associated loci discovered by the Immunochip array at a 99% confidence level^14^. RA loci, as defined from the Immunochip analysis, were extended to include the credible SNP region where necessary and overlapping regions were merged using the BEDTools v2.21.0 (ref. 22) merge command resulting in 211 associated regions.

### Target enrichment design

To remain hypothesis free and to validate significant findings, two target enrichments were designed. The first targeted the ‘associated region' and was called the ‘Region Capture' set. The second targeted all known gene promoters overlapping the region 500 kb up- and downstream of the lead disease-associated SNP dubbed as the ‘Promoter Capture' set. Capture oligos (120 bp; 25–65% GC, <3 unknown (N) bases) were designed using a custom Perl script within 400 bp but as close as possible to each end of the targeted HindIII restriction fragments and submitted to the Agilent eArray software (Agilent) for manufacture.

### Region Capture design

Capture oligonucleotides were designed to all HindIII restriction fragments in each previously defined associated region after excluding those already targeted in the Promoter Capture. Regions were extended by one restriction fragment where there was <500 bp between the restriction site and the region start/end. This resulted in 3,159 restriction fragments in total after merging overlapping regions. Of these, 1,028 failed design, 1,096 had both ends captured and 1,035 had one end captured, producing a target capture of 387.24 kb covering a genomic region of 7.46 Mb (3.5 kb/restriction fragment on average). In addition, a control region, which represents a well-characterized region of long-range interactions, was also included: *HBA* (174.57 kb genomic; 26 restriction fragments; 6.71 kb/restriction fragment).

### Promoter Capture design

Promoter Capture target regions were defined as 500 kb up- and downstream of each disease-associated SNP. These regions were further extended to encompass the associated regions where appropriate. HindIII restriction fragments were identified within 500 bp of the transcription start site of all genes mapping to the defined regions (Ensembl release 75; GRCh37) and overlapping regions were merged using the BEDTools^22^ merge command resulting in 6,296 restriction fragments. Of these, 792 failed design, 2,986 had both ends captured and 2,518 had one end captured, producing a target capture of 1.02 Mb. The 5,504 captured restriction fragments covered a genomic region of 38.76 Mb (7.04 kb/restriction fragment on average) and contained promoters for 3,857 genes. The *HBA* control region previously mentioned was also included.

### Cell culture and crosslinking

The GM12878 B-lymphoblastoid cell line, produced from the blood of a female donor with northern and western European ancestry by EBV transformation, was obtained from Coriell Institute for Medical Research. Lymphoblastoid cell lines were cultured in Roswell Park Memorial Institute (RPMI) 1640 per 20 mM L-glutamine supplemented with 15% foetal bovine serum (FBS) in 25 cm^2^ vented culture flasks at 37 °C per 5% CO_2_. The T-lymphoblastoid Jurkat E6.1 cell line, originating from the peripheral blood of a 14-year-old boy in the study by Schneider *et al.*^23^, was obtained from LGC Standards and cultured in RPMI 1640 per 20 mM L-glutamine supplemented with 10% FBS in 25 cm^2^ vented culture flasks at 37 °C/5% CO_2_. To generate Hi-C libraries, 5–6 × 10^7^ GM12878 and Jurkat cells were grown to ∼90% confluence then formaldehyde crosslinking was carried out as described in the study by Belton *et al.*^15^. Cells were washed in Dulbecco's Modified Eagle's medium (DMEM) without serum then crosslinked with 2% formaldehyde for 10 min at room temperature. The crosslinking reaction was quenched by adding cold 1 M glycine to a final concentration of 0.125 M for 5 min at room temperature, followed by 15 min on ice. Crosslinked cells were washed in ice-cold PBS, the supernatant discarded and the pellets flash-frozen in liquid nitrogen and stored at −80 °C.

### Hi-C library generation

Cells were thawed on ice and re-suspended in 50 ml freshly prepared ice-cold lysis buffer (10 mM Tris-HCl pH 8, 10 mM NaCl, 0.2% Igepal CA-630, one protease inhibitor cocktail tablet). Routinely, two pellets from each cell line were re-suspended and combined in 7 ml complete lysis buffer to give ∼5-6 × 10^7^ cells. Cells were lysed on ice for a total of 30 min, with 2 × 10 strokes of a Dounce homogeniser with a 5-min break between Douncing. Following lysis, the nuclei were pelleted and washed with 1.25 × NEB Buffer 2 then re-suspended in 1.25 × NEB Buffer 2 to make aliquots of 5–6 × 10^6^ cells for digestion. Following lysis, Hi-C libraries were digested using HindIII then prepared as described in the study by van Berkum *et al.*^24^ with modifications described in the study by Dryden *et al.*^6^. Pre-Capture amplification was performed with eight cycles of PCR on multiple parallel reactions from Hi-C libraries immobilized on Streptavidin beads, which were pooled post PCR and SPRI bead purified. The final library was re-suspended in 30 μl TLE and the quality and quantity assessed by Bioanalyzer and qPCR.

### Solution hybridization capture of Hi-C library

Hi-C samples corresponding to 750 ng were concentrated in a Speedvac then re-suspended in 3.4 μl water. Hybridization of SureSelect custom Promoter and Region Capture libraries to Hi-C libraries was carried out using Agilent SureSelectXT reagents and protocols. Post-capture amplification was carried out using six cycles of PCR from streptavidin beads in multiple parallel reactions, then pooled and purified using SPRI beads.

### Paired-end next generation sequencing

Two biological replicates for each of the cell lines were prepared for each target capture. Sequencing was performed on Illumina HiSeq 2500 generating 75 bp paired-end reads (Genomic Technologies Core Facility in the Faculty of Life Sciences, the University of Manchester). CASAVA software (v1.8.2, Illumina) was used to make base calls; reads failing Illumina filters were removed before further analysis. Promoter Capture libraries were each sequenced on one HiSeq lane and each Region Capture was sequenced on 0.5 of a HiSeq lane. Sequences were output in FASTQ format, poor quality reads truncated or removed as necessary, using Trimmomatic version 0.30 (ref. 25), and subsequently mapped to the human reference genome (GRCh37/hg19) and filtered to remove experimental artefacts using the Hi-C User Pipeline (HiCUP, http://www.bioinformatics.babraham.ac.uk/projects/hicup/). Off-target di-tags, where neither end mapped to a targeted HindIII restriction fragment, were removed from the final data sets using a combination of BEDTools and command line tools. Full details of the number and proportion of excluded di-tags are given in Supplementary Table 3.

### Analysis of Hi-C interaction peaks

Di-tags separated by <20 kb were removed prior to analysis, as 3C data have shown a very high-interaction frequency within this distance^26^. Di-tags were then assigned to one of the four categories of ligations defined in the study by Dryden *et al.*^6^ using custom scripts: (1) single baited, *cis* interaction (<5 Mb); (2) single baited *cis* interactions (>5 Mb); (3) double-baited *cis* and (4) *trans* (either single or double baited). Significant interactions for *cis* interactions within 5 Mb were determined using the ‘High resolution analysis of the *cis* interaction peaks' method described in the study by Dryden *et al*^6^. To correct for experimental biases, the interactability of each fragment was determined. Interactability is calculated from the interactions from a particular baited HindIII restriction fragment to long-range, ‘*trans*' fragments, under the assumption that those represent random, background interactions and so should be similar in any particular baited fragment. The resulting distribution is bimodal consisting of stochastic noise (low *trans* counts) and genuine signal (high *trans* counts). A truncated negative binomial distribution was fitted to the distribution with the negative binomial truncation point for interacting restriction fragments set at a count of 3,000 and non-interacting set at 1,500 for the Promoter Capture and 600 for the Region Capture due to differences in read depth. The 5% quantile point of the non-truncated distribution was determined to provide the noise threshold. For both cell lines in both captures, the noise threshold was determined to be 400 di-tags and therefore all restriction fragments with fewer than 400 di-tags were filtered out. A negative binomial regression model was fitted to the filtered data correcting for the interactability of the captured restriction fragment and interaction distance. For interactions, where both the target and baited region were captured (double-baited interactions), we also accounted for the interactability of the other end.

We wanted to examine whether concordance between interactions called in the Region and Promoter Capture experiments increased with decreasing FDR thresholds. This is complicated because we can only define the set of interactions that could have been observed in both experiments conditional on those that were observed at a given FDR threshold in one experiment. We therefore decided to normalize to those interactions called at an FDR threshold of 20% in the region experiment and defined the following enrichment parameter: *X*[*i*,*j*]=*P* (called in Region Capture at FDR *i* and in Promoter Capture at FDR *j*| called in Region Capture at FDR 20%)/*P*(called in Region Capture at FDR *i*| called in Region Capture at FDR 20%).

Interactions were considered statistically significant after combining replicates and filtering on FDR≤5%. Significant Interactions were visualized in the WashU Epigenome Browser (http://epigenomegateway.wustl.edu/browser/)^27^,^28^.

## Additional information

**Accession codes:** Raw data and HindIII restriction fragment interaction counts are available in the NCBI Gene Expression Omnibus (GEO; http://www.ncbi.nlm.nih.gov/geo/) under accession number GSE69600.

**How to cite this article:** Martin, P. *et al.* Capture Hi-C reveals novel candidate genes and complex long-range interactions with related autoimmune risk loci. *Nat. Commun.* 6:10069 doi: 10.1038/ncomms10069 (2015).

## Supplementary Material

Supplementary Information Supplementary Figures 1-7 and Supplementary Tables 1-3.

Supplementary Data 1 GM12878 Region Capture Interactions Validated at >50 Observed/Expected in Rao et al.
