# Supplementary material for: Capture Hi-C reveals novel candidate genes and complex long-range interactions with related autoimmune risk loci
Source: Nat Commun. 2015 Nov 30;6:10069. doi: 10.1038/ncomms10069 (PMC4674669; doi:10.1038/ncomms10069)
Supplement: Supplementary Information — Supplementary Figures 1-7 and Supplementary Tables 1-3. [file ncomms10069-s1.pdf]

a

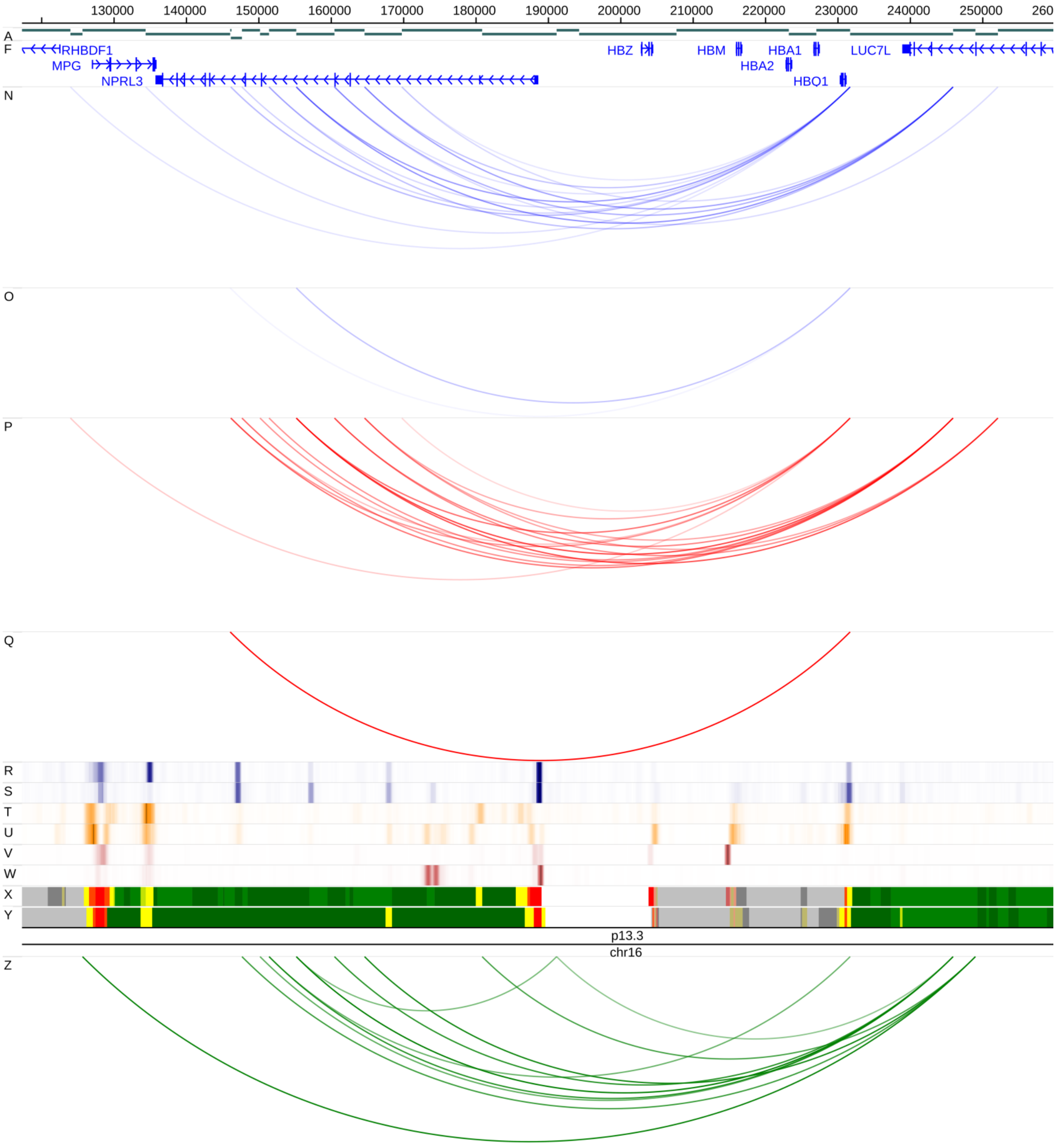

130000
140000
150000
160000
170000
180000
190000
200000
210000
220000
230000
240000
250000
260
A
F
RHBDF1
MPG
NPRL3
HBZ
HBM
HBA1
HBA2
HBQ1
LUC7L
N
O
P
Q
R
S
T
U
V
W
X
Y
p13.3
chr16
Z

b

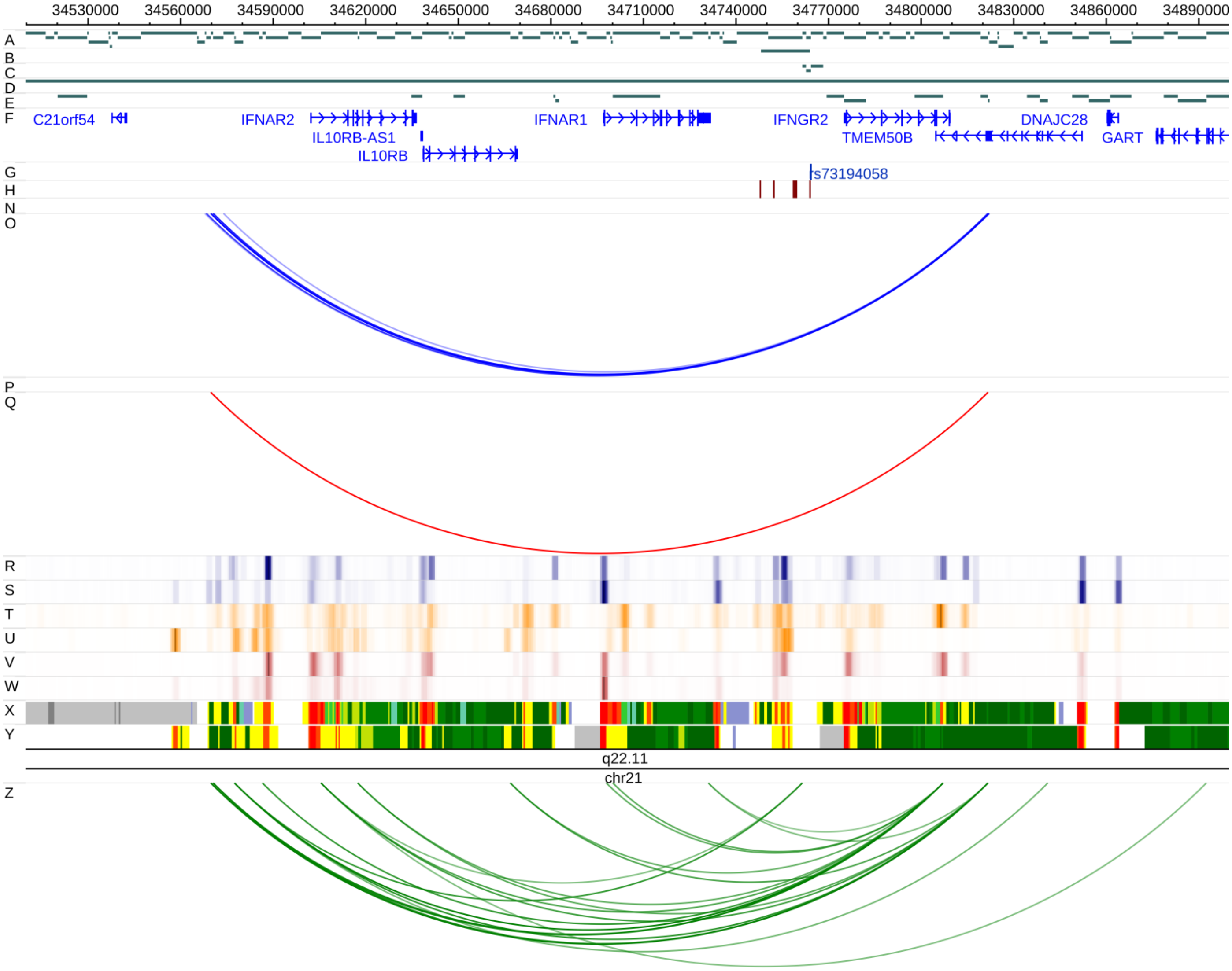
34530000
34560000
34590000
34620000
34650000
34680000
34710000
34740000
34770000
34800000
34830000
34860000
34890000
A
B
C
D
E
F
C21orf54
IFNAR2
IL10RB-AS1
IL10RB
IFNAR1
IFNGR2
TMEM50B
DNAJC28
GART
G
rs73194058
H
N
O
P
Q
R
S
T
U
V
W
X
Y
q22.11
chr21
Z

c

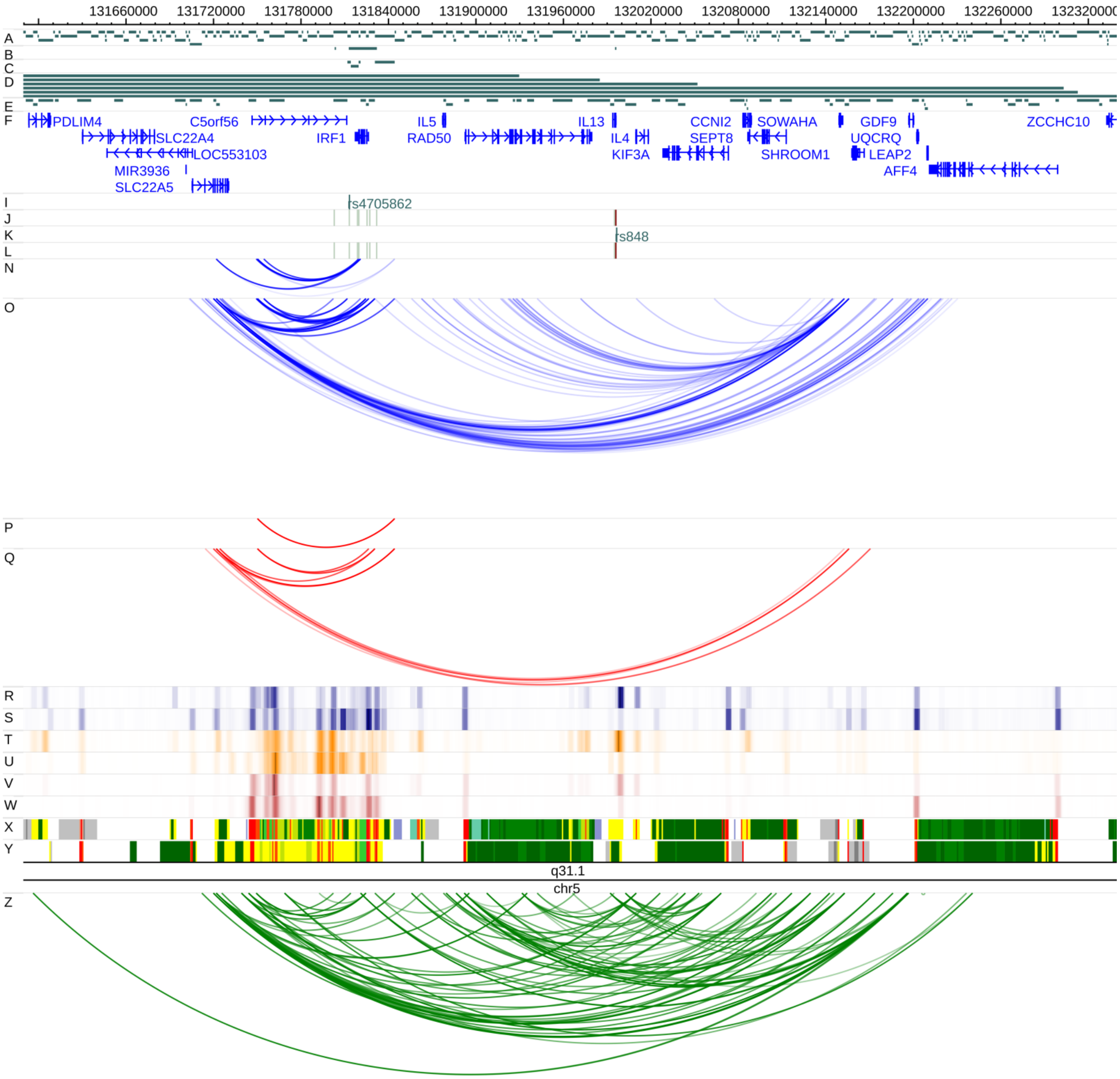

Supplementary Figure 1 Comparison to previously published ENCODE UMass 5C data. Both GM12878 and Jurkat cell lines replicate previously observed interactions at the *HBA* (**a**), *IFNAR1* (**b**) and *IL5* (**c**) loci. Genomic co-ordinates are shown along the top of each panel and tracks are labelled A-Y (empty tracks removed for clarity): A – HindIII restriction fragments; B-E – Regions targeted and restriction fragments included in the region (B, C) and promoter (D, E) capture experiments; F – RefSeq Genes from the UCSC Genome Browser, downloaded Jan 1, 2012; G,I,K – Index SNPs identified for RA (G), JIA (I) and PsA (K). Associations in red were identified in the RA Immunochip study. SNPs in blue were novel associations identified in the RA trans-ethnic GWAS meta-analysis, JIA and PsA SNPs were identified in the JIA and PsA Immunochip studies; H,J,L – Density plots showing 1000 Genomes SNPs in LD ($r2\geq0.8$) with the index SNPs (green-red) for RA (H), JIA (J) and PsA (L); M – T1D Credible set SNPs identified in the T1D Immunochip study; N,O – Significant Interactions identified in the region and promoter capture experiments in GM12878 (N, O) and Jurkat (P, Q) cells; R-Y – Data from the WashU Encode track hub showing DNaseI HS sites, H3K4me1 histone marks and H3K27ac histone marks for GM12878 (R, T, V) and CD3 Primary (S, U, W) cells and BROAD ChromHMM states for GM12878 (X) and CD4 Naive Primary cells (Y); Z – Long range interaction data from ENCODE UMass 5C data for GM12878 cells.

a

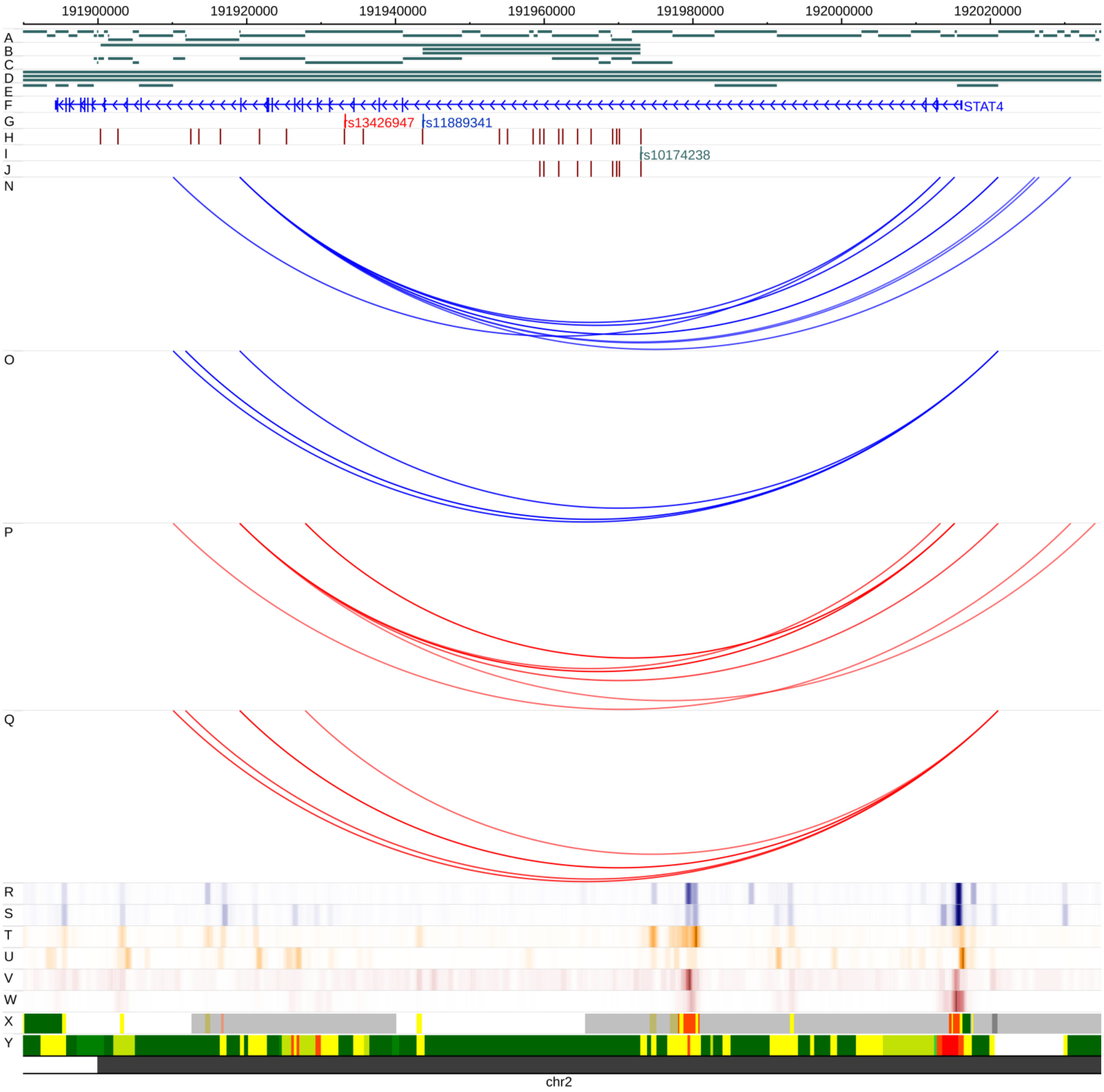

191900000
191920000
191940000
191960000
191980000
192000000
192020000
A
B
C
D
E
F
G
H
I
J
N
O
P
Q
R
S
T
U
V
W
X
Y
STAT4
rs13426947
rs11889341
rs10174238
chr2

b

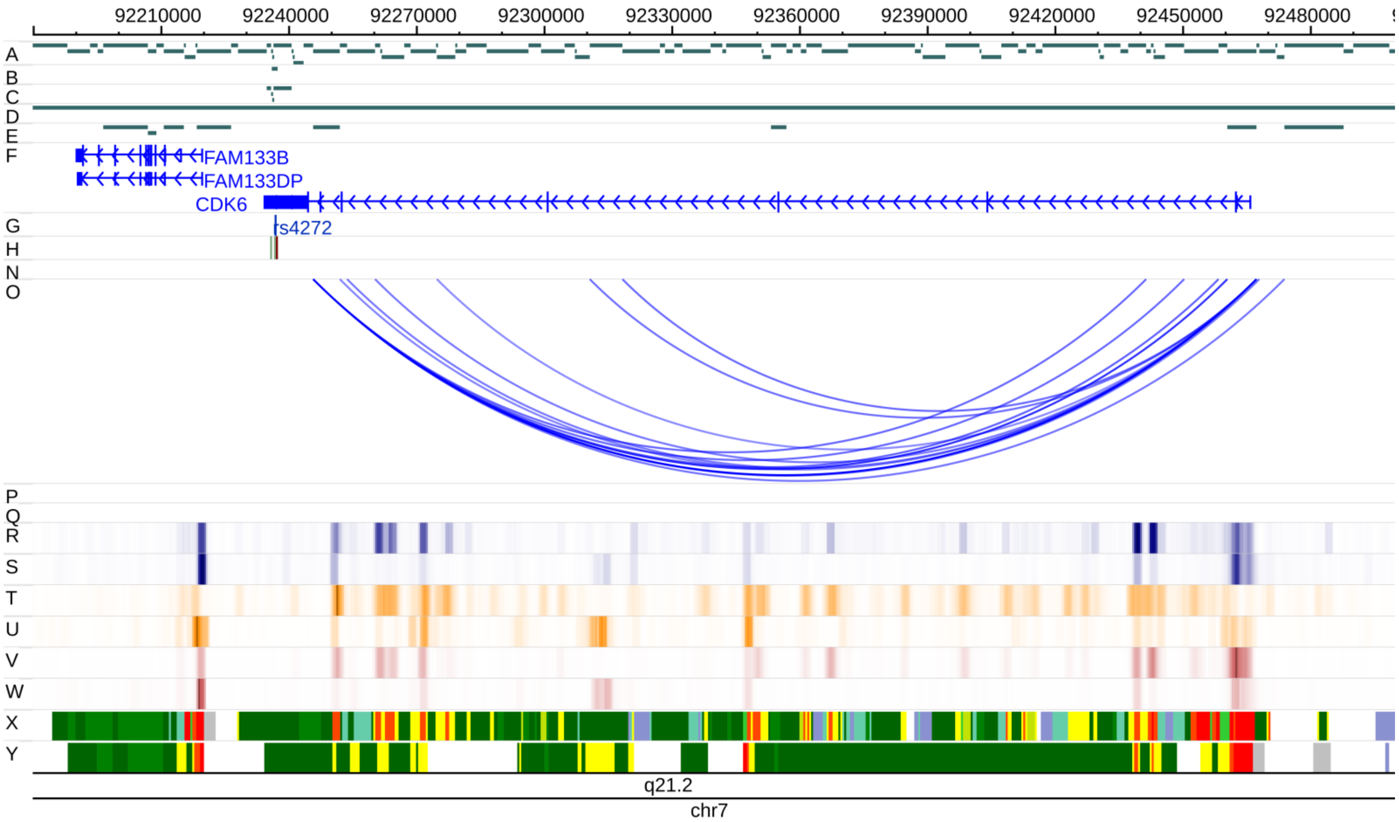

Supplementary Figure 2 Examples of Interactions between a Gene Promoter and Associated SNPs within the Same Gene. (**a**) SNPs located in the introns of *STAT4* show interactions with the *STAT4* promoter. (**b**) SNPs in the 3' UTR of *CDK6* show interaction with the *CDK6* promoter in GM12878 cells. Genomic co-ordinates are shown along the top of each panel and tracks are labelled A-Z as **Supplementary Figure 1**.

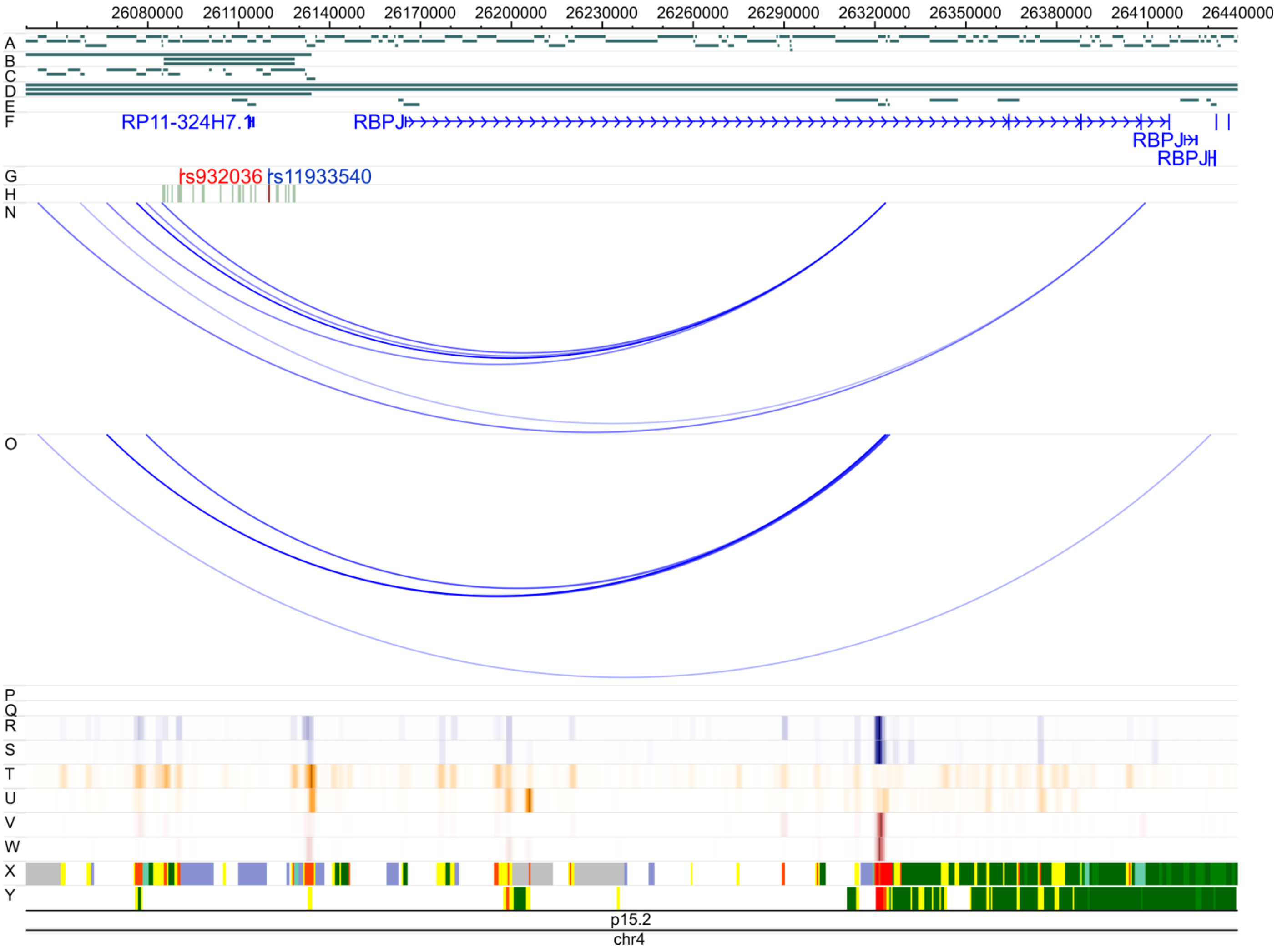

Supplementary Figure 3 RBPJ Locus. Associated SNPs within a lncRNA show interaction with the *RBPJ* gene promoter in GM12878 cells. Genomic co-ordinates are shown along the top of each panel and tracks are labelled A-E and G-Z as **Supplementary Figure 1**. Track F has been replaced with the GENCODE V17 genes to show the lncRNA.

a

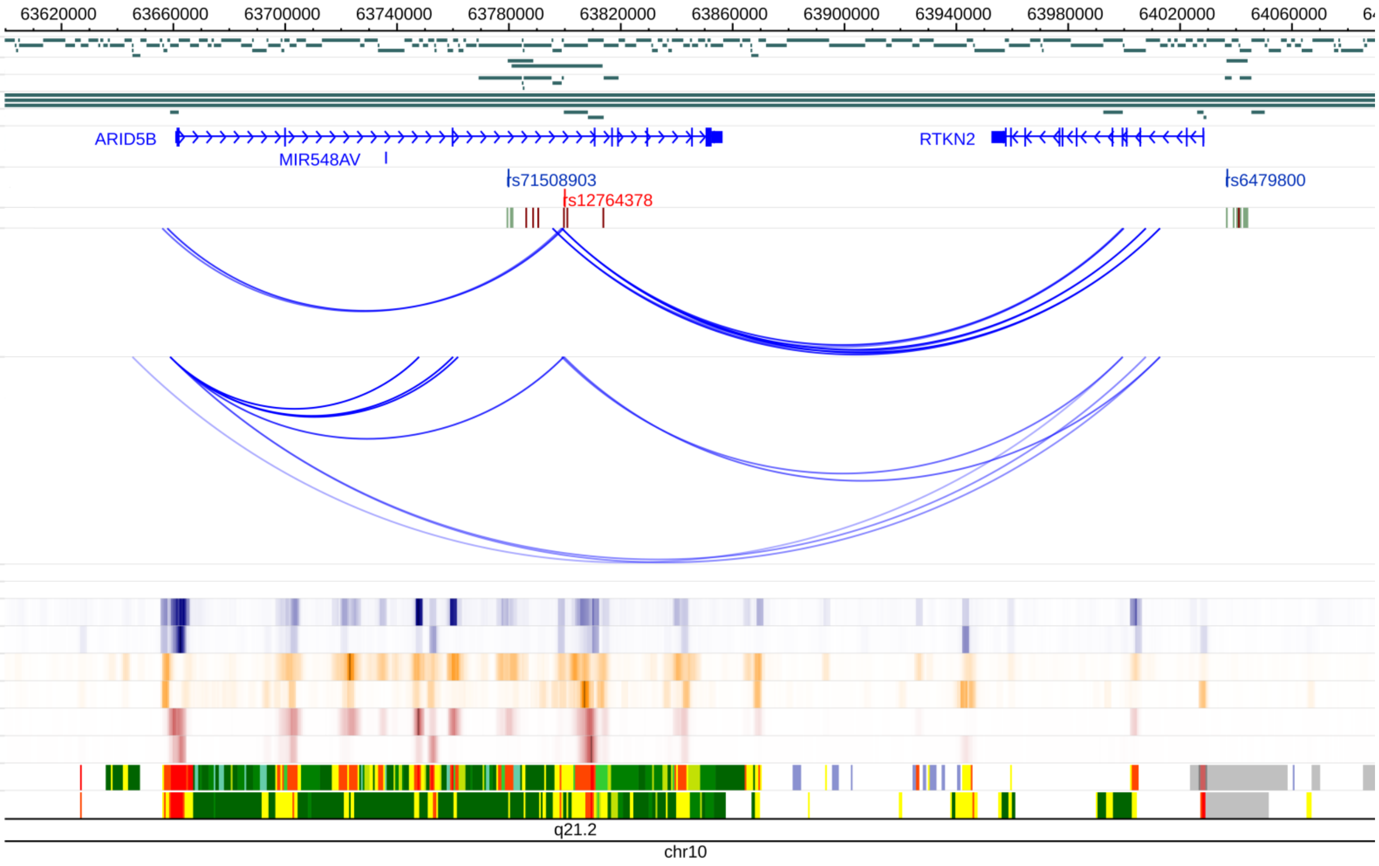

63620000
63660000
63700000
63740000
63780000
63820000
63860000
63900000
63940000
63980000
64020000
64060000
A
B
C
D
E
F
G
H
N
O
P
Q
R
S
T
U
V
W
X
Y
ARID5B
MIR548AV
RTKN2
rs71508903
rs12764378
rs6479800
q21.2
chr10

b

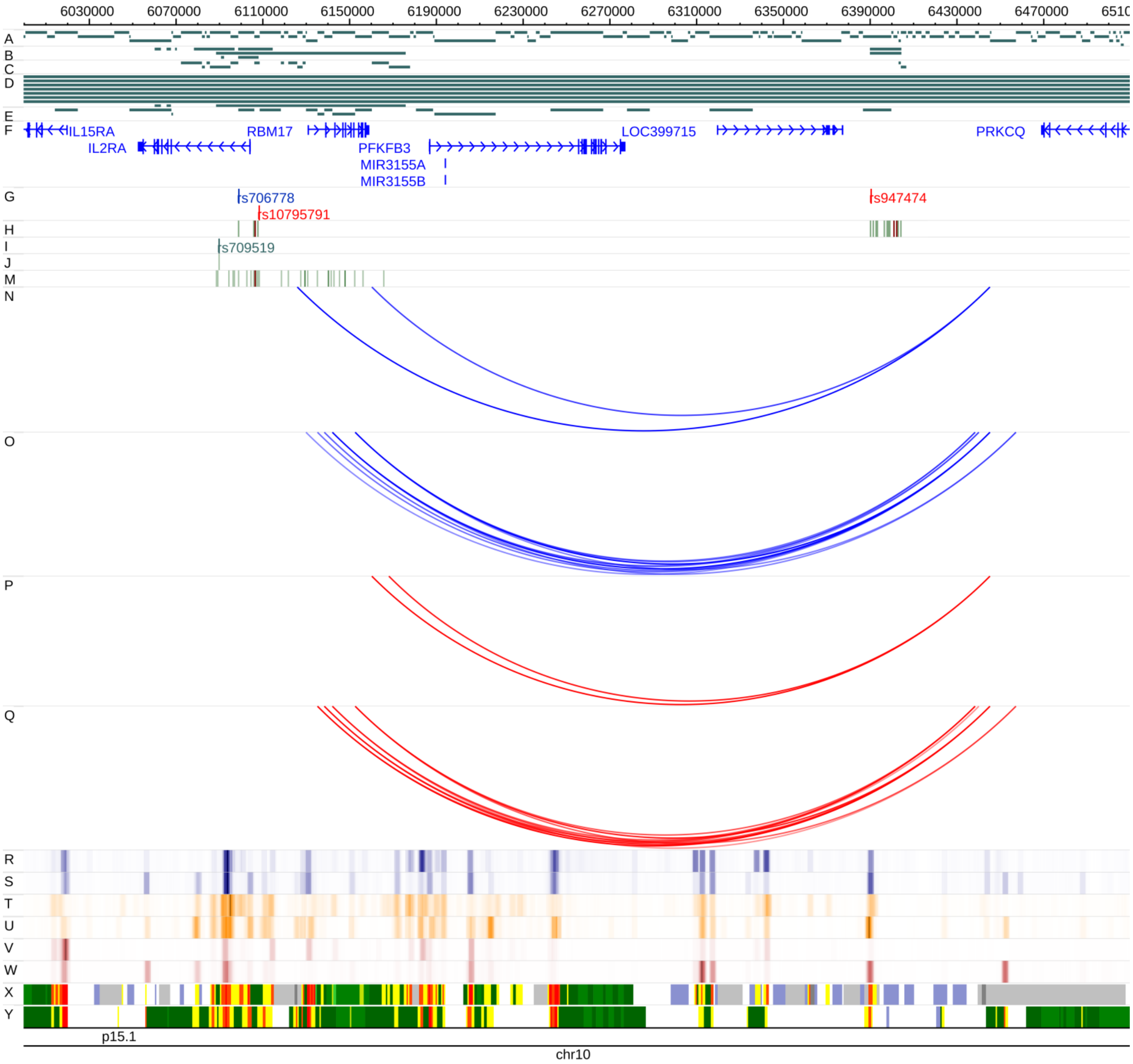
6030000
6070000
6110000
6150000
6190000
6230000
6270000
6310000
6350000
6390000
6430000
6470000
A
B
C
D
E
F
G
H
I
J
M
N
O
P
Q
R
S
T
U
V
W
X
Y
IL15RA
IL2RA
RBM17
PFKFB3
MIR3155A
MIR3155B
LOC399715
PRKCQ
rs706778
rs10795791
rs947474
rs709519
p15.1
chr10

c

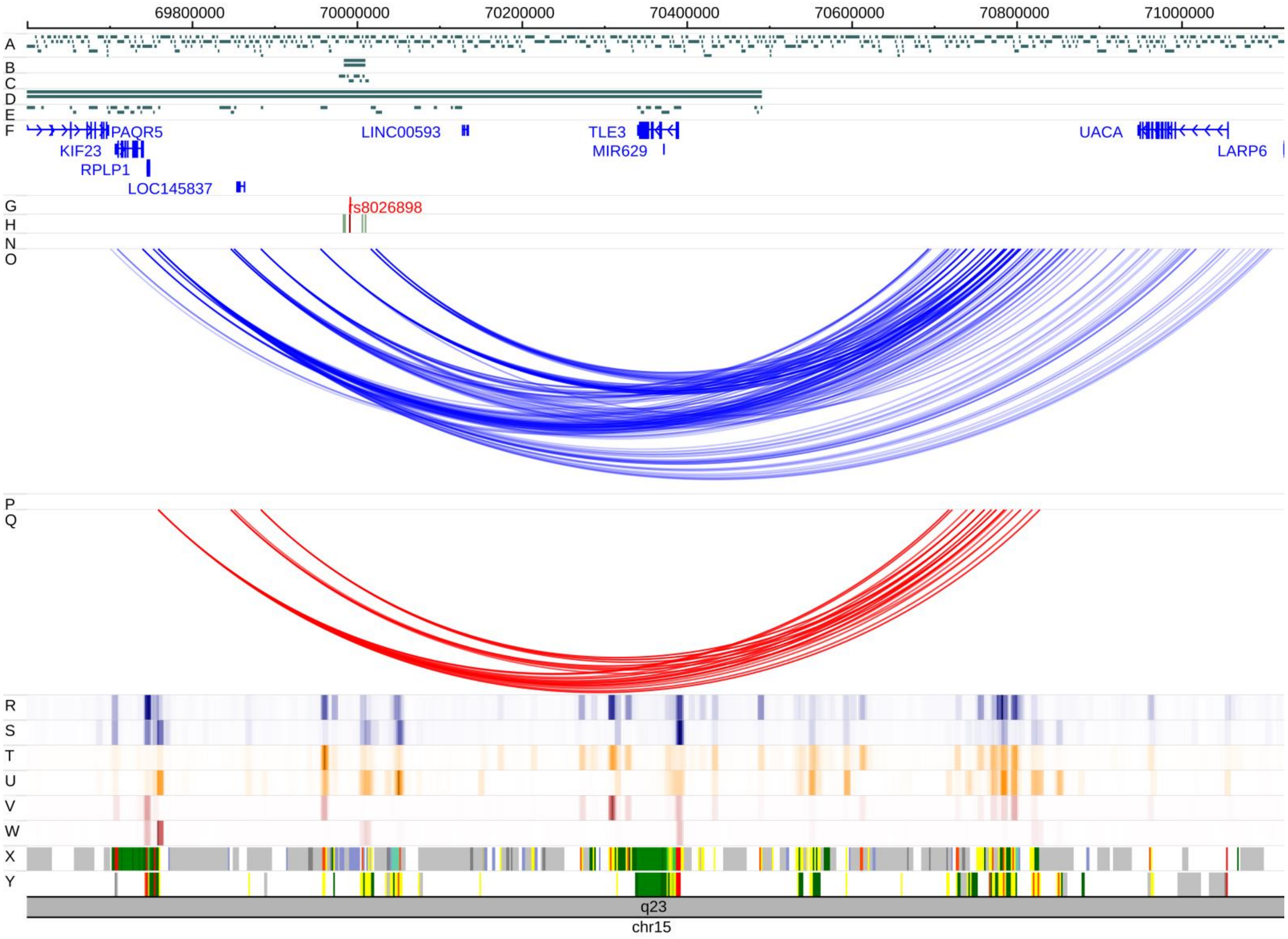

Supplementary Figure 4 Examples of long range interactions. Restriction fragments proximal to disease associated SNPs show interaction with gene promoters some distance away. (**a**) *ARID5B* Locus – SNPs located within an intron of *ARID5B,* shown to interact with the promoter of *ARID5B*, also display a long range interaction with *RTKN2*. (**b**) *IL2RA* Locus – SNPs located upstream of *PRKCQ* display interaction to the *IL2RA* gene promoter. (**c**) *TLE3* Locus – SNPs upstream of *TLE3* display long range interaction with the *UACA* and *LARP6* loci. Genomic co-ordinates are shown along the top of each panel and tracks are labelled A-Z as **Supplementary Figure 1**.

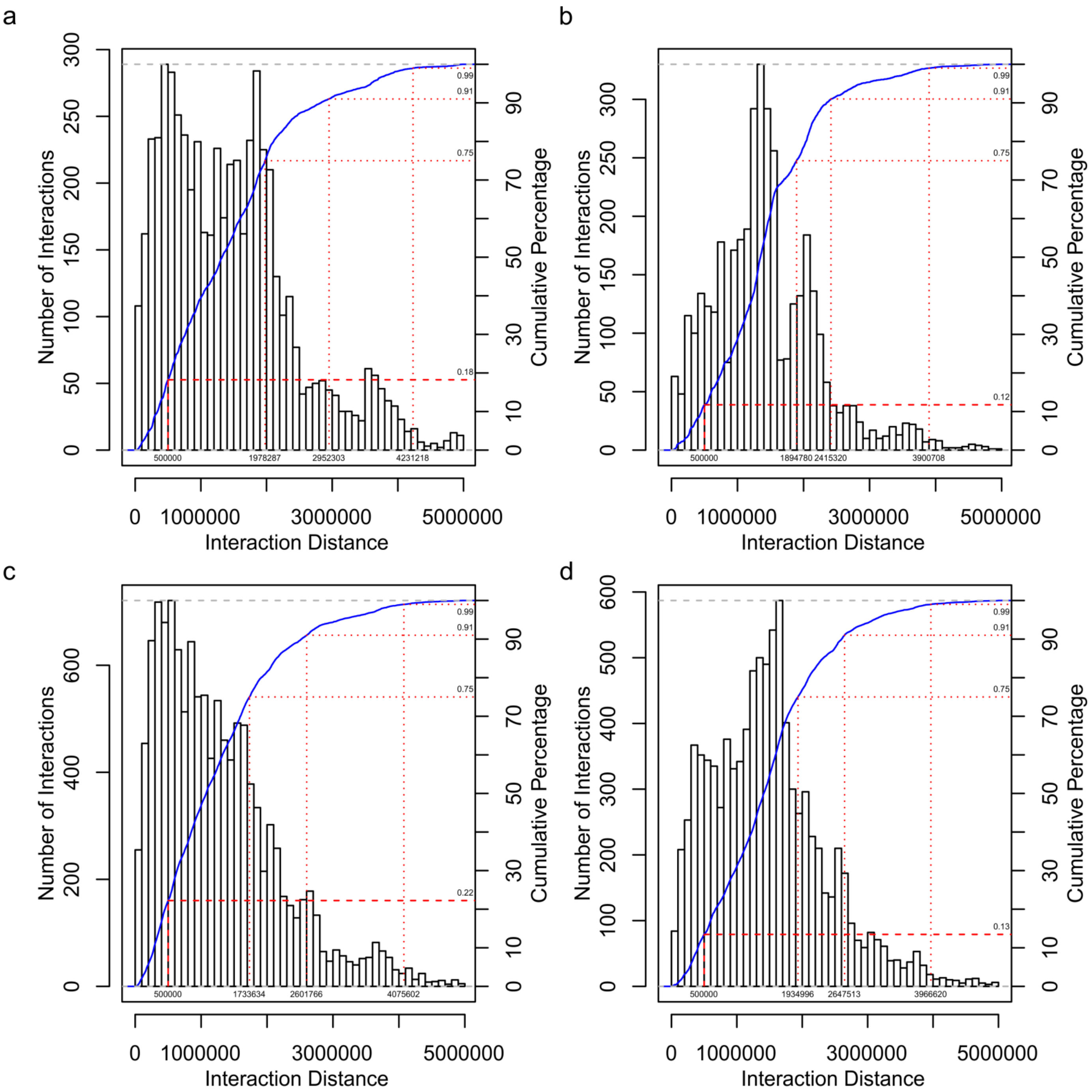

Supplementary Figure 5 Interaction Distance Histograms. The number of interactions are shown for 10Kb bins up to 5Mb for region and promoter capture experiments in GM12878 (**a**, **c**) and Jurkat (**b**, **d**) cells. Blue lines indicate the cumulative percentage of interactions, dotted red lines mark the 75th, 91st and 99th centiles and dashed red lines indicate the percentage of interactions ≤500,000.

a

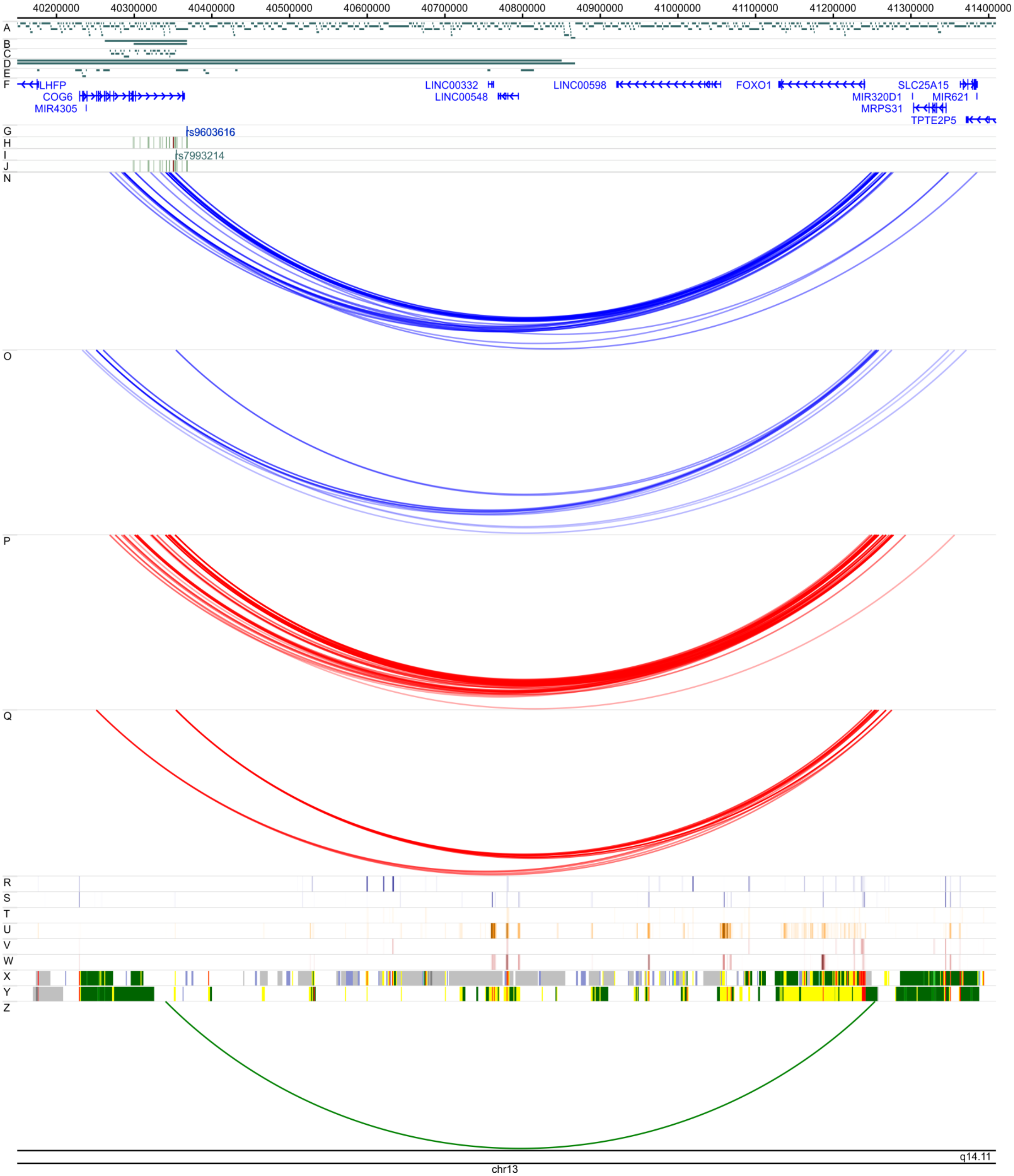
40200000
40300000
40400000
40500000
40600000
40700000
40800000
40900000
41000000
41100000
41200000
41300000
41400000
A
B
C
D
E
F
LHFP
COG6
MIR4305
LINC00332
LINC00548
LINC00598
FOXO1
MIR320D1
MRPS31
SLC25A15
MIR621
TPTE2P5
G
H
I
J
rs9603616
rs7993214
N
O
P
Q
R
S
T
U
V
W
X
Y
Z
q14.11
chr13

b

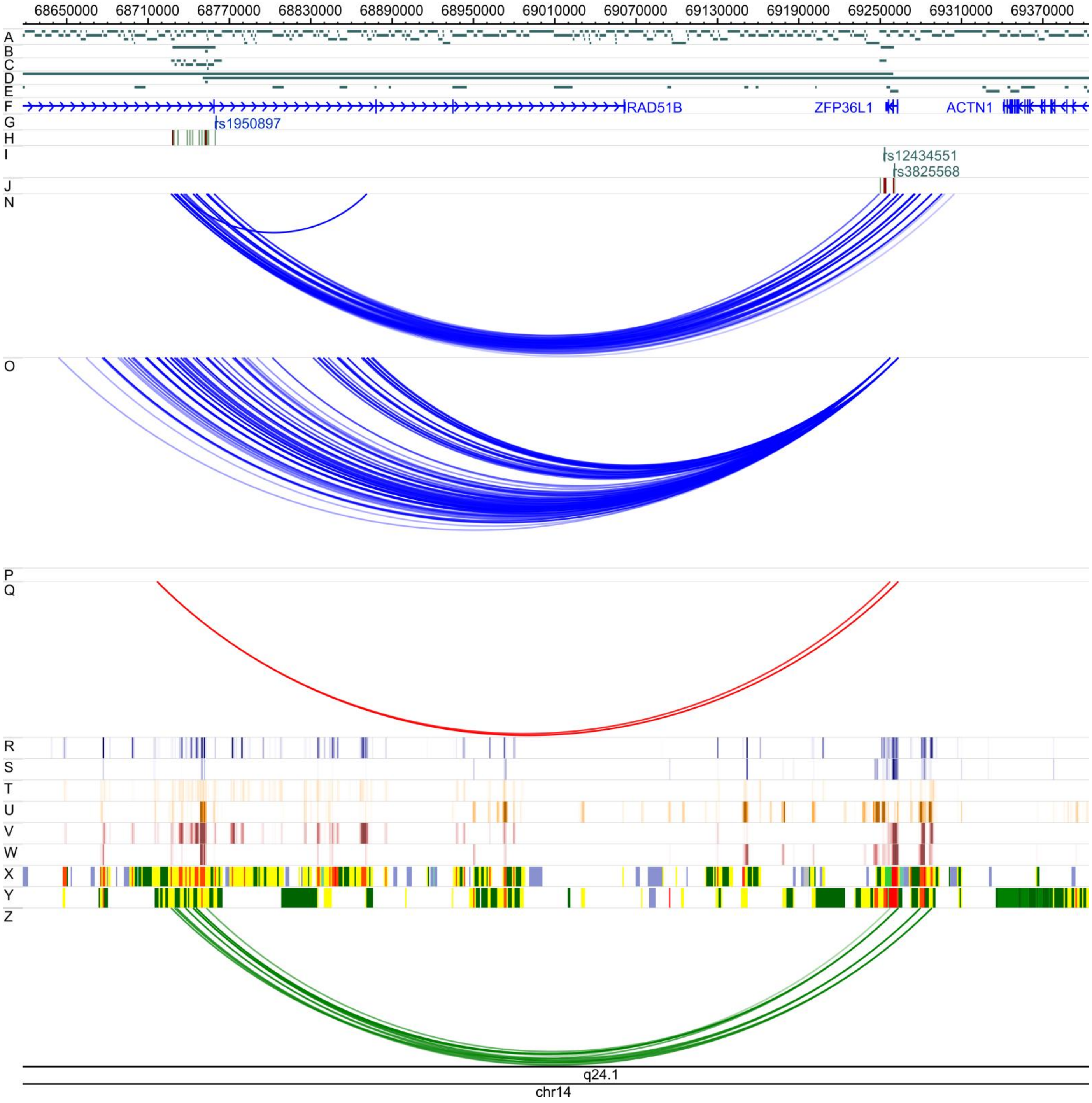

Supplementary Figure 6 Interactions Validated in Promoter Capture and Rao *et al.* Hi-C data. (**a**) *COG6*/*FOXO1* Locus - Interactions > 500kb between SNPs located in the *COG6* gene and the *FOXO1* gene previously seen in the complement Promoter Capture are also observed in the Rao *et al.* Hi-C data at 50X observed/expected. (**b**) *ZFP36L1* Locus - Interactions >500kb between SNPs associated with RA and JIA with the *ZFP36L1* gene previously seen in the complement Promoter Capture are also observed in the Rao *et al.* Hi-C data at 50X observed/expected. Genomic co-ordinates are shown along the top of each panel and tracks are labelled A-Y as **Supplementary Figure 1**. Track Z shows data from Rao *et al.* in GM12878 cells at >50X observed/expected.

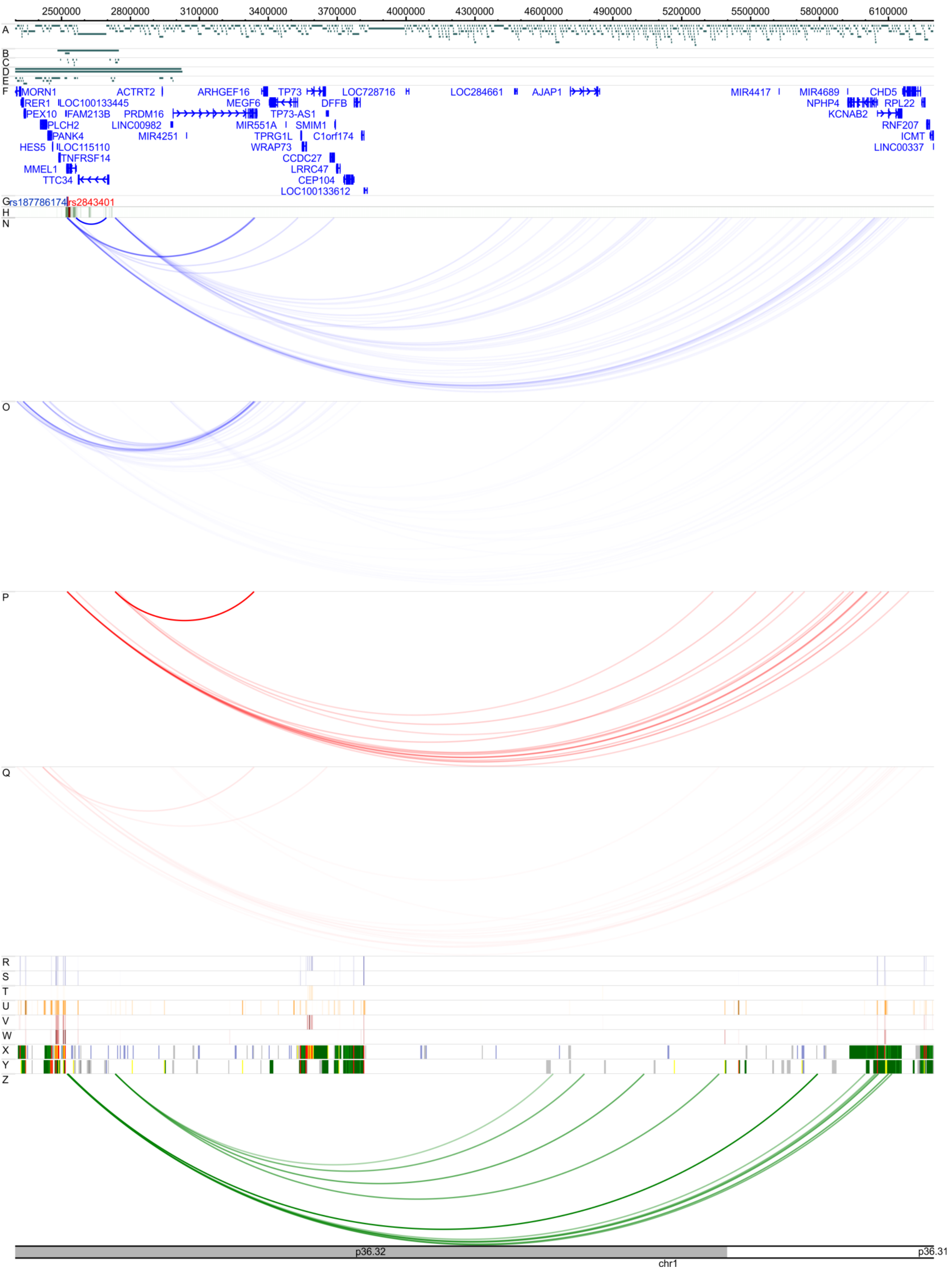

Supplementary Figure 7 Interactions Validated in Rao *et al.* Hi-C data - *MMEL1* Locus. SNPs located near to the *MMEL1* gene are shown to interact with multiple sites greater than 2.1Mb away in both the Region Capture and the Rao *et al.* Hi-C data in GM12878 cells. Genomic co-ordinates are shown along the top of each panel and tracks are labelled A-Y as Supplementary Figure 1. Track Z shows data from Rao *et al.* in GM12878 cells at >50X observed/expected.

Supplementary Table 1 Validated Regions By Disease

Region Disease	Region	Gene(s) Implicated By Capture Hi-C	SNPs involved in interaction(s)	SNP Disease
T1D	chr1:172668340-172793418	C1orf105,CENPL|DARS2, RNU6-693P,SUCO,TNFSF4		
T1D	chr17:38753550-38861757	EIF1	rs113561632,rs11658582,rs7224919,rs7226035	T1D
RA/T1D	chr2:204691538-204745003|rs3087243|rs3087243|rs11571302	RAPH1	rs13030124,rs13089409,rs1968351,rs34636506,rs6745050	T1D
			rs13030124,rs144115812,rs1968351,rs34636506,rs6745050	RA
T1D/JIA	chr3:46150937-46541541|rs62625034	CCR5	rs112590754,rs62625034	T1D
		CXCR6	rs11575821,rs746492,rs7652037	T1D
RA/T1D	chr4:26031095-26134258|rs11933540|rs932036	RBPJ		
T1D	chr5:35800547-35927309	SPEF2	rs11955657,rs11955690,rs34442743,rs3734107,rs3866449, rs3866450,rs4024109,rs4024110,rs6873195,rs72742471	T1D
JIA	6q23*	HBS1L		
		MYB		
PsA	rs111825814	RN7SL635P		
RA/JIA	rs13426947|rs11889341|rs10174238	STAT4	rs11893432, rs3024859	RA
JIA	rs1408272|6p22*	HFE,HIST1H4C		
JIA	rs1474348|7p15*	AC002480.3		
JIA	rs1800623|10q23*	CH25H		
RA/T1D	rs1950897|chr14:68752593-68754593	ZFP36L1	rs10131490,rs10551501,rs17105278,rs1885013,rs1950897, rs1957570,rs2104047,rs2208397,rs28498223,rs3784099, rs7148416,rs7148882,rs8008961,rs8015139,rs911263	RA
			rs12889006,rs2236262,rs3825568	JIA
RA	rs28411352	SF3A3|RNU6-510P	rs56023437	RA
RA	rs35677470|rs73081554	PXK		
RA	rs3783782	HIF1A	rs146492555,rs17098394,rs2031410,rs3783782,rs3783795, rs3783796,rs57887308,rs58360439,rs59149393,rs74450101, rs76199319,rs76644792,rs77812793,rs79882624	RA
		RP11-618G20.1	rs117584363,rs146492555,rs17098394,rs17098433,rs17098496, rs2031410,rs3783769,rs3783782,rs3783788,rs3783789, rs3783795,rs3783796,rs57887308,rs58360439,rs59149393, rs74450101,rs76199319,rs76269868,rs76644792,rs77812793, rs79882624	RA
JIA/PsA	rs4705862	SLC22A5	rs2548998	JIA/PsA
PsA	rs4936059	RP11-702B10.2	rs7126028,rs7126250	PsA
RA	rs570676|rs331463	COMMD9	rs5030485,rs5030486,rs5030487	RA
JIA	19p13*	LRRC25|AC008397.1		
		PDE4C		
RA	rs624988|rs798000	RP11-27K13.3|TTF2		
RA/PsA	rs657075|rs7703009|rs715285	RAPGEF6		
RA	rs71508903|rs12764378	RTKN2		
PsA	rs730086	CNP	rs730086	PsA
RA/PsA	rs73013527|rs4936059	RP11-702B10.2	rs10893901,rs11221402,rs1893866,rs4936059,rs7106876, rs7122004	PsA
			rs73013527	RA
RA/PsA	rs7752903|rs610604	AL357060.1,RP11-95M15.2,Y_RNA	rs654874	PsA
		RP11-10J5.1|RP11-240M16.1		
RA	rs9372120	PRDM1	rs2299864,rs9372120,rs9386516	RA
		PRDM1|RP1-134E15.3	rs2299864,rs9372120	
RA	rs9373594	RP11-162J8.3,UST		
RA	rs9826828	MSL2,PCCB		

* personal communication

Supplementary Table 2 Region Statistics

Experiment		Cell	
		GM12878	Jurkat
Region Capture	Average Region Size (bp)	64,787	
	Average Number of Interactions per Region	48.7	38.2
	Average Number Interactions per Restriction Fragment	8.9	8.3
	Average Interaction Distance (bp)	1,451,825	1,430,506
	Number of Regions Interacting with a Promoter	37	25
	Number of Fragments Interacting with a Promoter	136	63
	Number of Associated SNPs in the Interacting Regions ($r^2 \geq 0.8$)	5,329	
	Number of Associated SNPs in the Interacting Fragments ($r^2 \geq 0.8$)	671	449
	Number of Associated SNPs in the Interacting Regions ($r^2 \geq 0.9$)	3,033	
	Number of Associated SNPs in the Interacting Fragments ($r^2 \geq 0.9$)	504	292
Promoter Capture	Average Number of Genes in 1Mb Regions	359	
	Average Distance of Promoters to Region	212,762	
	Number of Genes that show Interactions	1,341	1,136
	Average Interaction Distance (bp)	1,262,173	1,472,446

Supplementary Table 3 Sequencing & QC Statistics

Cell	Capture Experiment	Replicate	Raw Reads	QC Filtered Reads	Aligned Pairs	Valid Di-tags	Unique Di-tags	Cis	Trans	Cis/Trans	On Target (%)	On Target Cis	On Target Trans	On Target Cis/Trans	Bait to Bait (%)
GM12878	Region	Rep 1	80,014,978	78,467,719	59,929,732	51,465,358	45,892,434	38,853,120	7,039,314	5.52	20,113,771 (43.83%)	17,013,285	3,100,486	5.49	3,359,680 (16.7%)
GM12878	Region	Rep 2	112,175,016	108,735,810	88,841,743	77,796,593	46,255,464	37,792,853	8,462,611	4.47	40,793,883 (88.19%)	33,366,346	7,427,537	4.49	6,199,018 (15.2%)
Jurkat	Region	Rep 1	78,543,748	76,963,038	60,126,486	52,225,839	44,810,487	37,562,253	7,248,234	5.18	28,073,973 (62.65%)	23,541,206	4,532,767	5.19	5,877,743 (20.94%)
Jurkat	Region	Rep 2	105,436,603	101,765,348	77,022,176	69,953,227	50,891,861	41,540,063	9,351,798	4.44	26,829,286 (52.72%)	21,840,131	4,989,155	4.38	3,173,044 (11.83%)
GM12878	Promoter	Rep 1	151,946,987	148,618,189	110,497,620	94,604,772	81,500,754	68,047,789	13,452,965	5.06	46,738,753 (57.35%)	38,688,459	8,050,294	4.81	4,917,795 (10.52%)
GM12878	Promoter	Rep 2	186,043,905	181,179,872	137,965,848	119,415,709	85,847,090	68,580,262	17,266,828	3.97	74,373,047 (86.63%)	59,359,899	15,013,148	3.95	7,411,636 (9.97%)
Jurkat	Promoter	Rep 1	159,267,590	155,743,979	116,474,859	100,827,324	85,111,648	70,572,012	14,539,636	4.85	59,494,411 (69.9%)	49,187,395	10,307,016	4.77	4,173,962 (7.02%)
Jurkat	Promoter	Rep 2	177,413,627	172,658,827	126,626,485	114,899,145	86,965,933	70,383,387	16,582,546	4.24	55,491,937 (63.81%)	44,699,709	10,792,228	4.14	5,073,930 (9.14%)
